# EVA: Exome Variation Analyzer, an efficient and versatile tool for filtering strategies in medical genomics

**DOI:** 10.1186/1471-2105-13-S14-S9

**Published:** 2012-09-07

**Authors:** Sophie Coutant, Chloé Cabot, Arnaud Lefebvre, Martine Léonard, Elise Prieur-Gaston, Dominique Campion, Thierry Lecroq, Hélène Dauchel

**Affiliations:** 1 University of Rouen, INSERM U1079 Molecular genetics of cancer and neuropsychiatric diseases, 76183 Rouen cedex, France; 2 University of Rouen, LITIS EA 4108 Computer science, information processing and systems laboratory, 76821 Mont-Saint-Aignan cedex, France; 3 Institute of Research and Biomedical Innovation (IRIB), Haute-Normandie, France

## Abstract

**Background:**

Whole exome sequencing (WES) has become the strategy of choice to identify a coding allelic variant for a rare human monogenic disorder. This approach is a revolution in medical genetics history, impacting both fundamental research, and diagnostic methods leading to personalized medicine. A plethora of efficient algorithms has been developed to ensure the variant discovery. They generally lead to ~20,000 variations that have to be narrow down to find the potential pathogenic allelic variant(s) and the affected gene(s). For this purpose, commonly adopted procedures which implicate various filtering strategies have emerged: exclusion of common variations, type of the allelics variants, pathogenicity effect prediction, modes of inheritance and multiple individuals for exome comparison. To deal with the expansion of WES in medical genomics individual laboratories, new convivial and versatile software tools have to implement these filtering steps. Non-programmer biologists have to be autonomous combining themselves different filtering criteria and conduct a personal strategy depending on their assumptions and study design.

**Results:**

We describe EVA (Exome Variation Analyzer), a user-friendly web-interfaced software dedicated to the filtering strategies for medical WES. Thanks to different modules, EVA (i) integrates and stores annotated exome variation data as strictly confidential to the project owner, (ii) allows to combine the main filters dealing with common variations, molecular types, inheritance mode and multiple samples, (iii) offers the browsing of annotated data and filtered results in various interactive tables, graphical visualizations and statistical charts, (iv) and finally offers export files and cross-links to external useful databases and softwares for further prioritization of the small subset of sorted candidate variations and genes. We report a demonstrative case study that allowed to identify a new candidate gene related to a rare form of Alzheimer disease.

**Conclusions:**

EVA is developed to be a user-friendly, versatile, and efficient-filtering assisting software for WES. It constitutes a platform for data storage and for drastic screening of clinical relevant genetics variations by non-programmer geneticists. Thereby, it provides a response to new needs at the expanding era of medical genomics investigated by WES for both fundamental research and clinical diagnostics.

## Background

Next-generation sequencing (NGS) technologies are widely used to answer key biological questions at the scale of the entire genome and with an unprecedented depth [1-4]. Whether determining genetic or genomic variations, cataloguing transcripts and assessing their expression levels, identifying DNA-protein interactions or chromatin modifications, surveying the species diversity in an environmental sample, all these tasks are now tackled with large-scale sequencing and require computer intensive bioinformatic analyses [5-7], although different.

Identification of genetic variations can be addressed by whole genome sequencing (WGS) or whole exome sequencing (WES) of single individuals. WGS is particularly attractive because it allows to access the full spectrum of genetic variations, i.e. coding and non coding Single Nucleotide Variations (SNV) and short insertion-deletion variants (indels), as well as Copy Number Variants (CNV) and Structural Variants (SV) [2,8]. In practice, out of major genome centers and *a fortiori *for the clinical routine translation, the development of this approach is still constrained by various difficulties such as the production organization, the yet expensive cost, the actual error rate of the technologies (~ 1 error per 100 kb; ~30, 000 erroneous variant calls for the whole genome), the sheer volume of data to store and to transfer, requiring intensive informatics infrastructures and robust bioinformatics and filter procedures to retain only clinically relevant variants [8,9]. As new genomes are sequenced, for example in the context of large projects like the 1000 Genomes Project [10], the number of expected variations may decrease. But, first complete individual constitutional genome sequencing studies reported 3-4 million of SNP per genome, 80-90% of which highly overlapped the National Center for Biotechnology Information public SNP database (dbSNP) [11], leaving anyway 0.5 million novel variations to sift per genome [8].

While WGS remains an appealing ultimate perspective, WES focusing on only the coding regions of the genome, has become in a few years the choice strategy to meet the challenge of identifying a coding allelic variant for rare human monogenic disorder [12]. Thanks to DNA enrichment techniques, targeted sequencing of coding regions decreases the cost and improves the efficiency of large-scale coding variations discovery compared with what would require the entire human genome. The human exome, made of ~180,000 exons for a size of ~30 Mbp, is 1.5% of the total human genome. Thereby, not only targeted selection strategy reduces the cost but also accelerates the discovery of coding genetic variants that cause rare Mendelian diseases. In 2009, Ng *et al. *[13], by using an intersection recurrence strategy, showed the proof of the concept that identifying a gene responsible for a rare dominantly inherited disorder (Freeman-Sheldon syndrome) was possible using WES of independant index cases. Since then, more and more papers confirmed the success of this strategy [14-17].

Up to now, classical approaches such as linkage analysis using genetic markers have been extensively used to identify the molecular basis for nearly 3,500 Mendelian disorders [18]. But for over 3,500 Mendelian disorders, the gene remains unknown [18,19]. The limited number of patients for rare diseases or the limited access to the related members of the family has been a frequent obstacle to conduct linkage analysis [14]. As the NGS technologies have emerged, the long and fastidious classical linkage analysis for human Mendelian disorders will be replaced by more direct identification of the causal variation(s) and the corresponding gene. Moreover, in numerous cases there are no caryotypic nor CGH-array anomaly or negative result with Sanger sequencing on known mutated genes or on neighbor genes in a pathway of interest, because of the low depth of this first generation sequencing technology [20]. So, the exome-scale sequencing approach generates a technological breakthrough in medical genetics history in fundamental research for disease gene discovery and consequently in terms of new diagnostic methods and personalized medicine [12,14,16,21].

Numerous algorithms and software tools have been developed to efficiently manage terabytes of raw sequence variation data from WES. Commonly adopted variation discovery pipelines include successive bioinformatics steps for quality control of the short reads, alignment of the short reads to a reference sequence, variation calling and variation annotation [1,19,22-24]. Generally, ~20,000 variations per individual exome are obtained. The challenge remains in efficient filtering strategies to find the causal variant(s) and corresponding gene for a rare disease, among these thousands of candidates. With this aim, additional analytical procedures which implicate various heuristic filtering strategies have emerged [19,24]. Usually, wide range common variations (more than 90% of the total) are firstly excluded. This is done by comparison to publicy available databases of human genetic variations and privately available variants from other exome sequencing projects. To narrow down the search on remaining variations (often between 200 to 500), other filters take into account the type of variations (focus on presumed deleterious allelic variants, i.e. nonsynonymous, nonsense, stop loss, frameshift, splice site) and evaluate the functional effect of variations on gene products. Usually, various criteria are inspected for this task such as the physical properties of the wild-type and variant amino acids, the structural properties affecting protein dynamics and stability, the integrity of functional motifs and binding domains or sites implicated to posttranslational processing and cellular localization of proteins, evolutionary properties derived from a sequence alignment [21-24]. Beside these molecular nature and effects of the alternative allelic variants, filtering strategies also have to take into account the mode of inheritance of the disorder suggested by pedigree (recessive or dominant model for Mendelian disorders or sporadic cases). Finally, taking advantages of multiple individuals, intersection or differential exome strategies can drastically reduce the remaining variations to several genes.

As the exome-scale sequencing is today positioned as a method of choice for disease gene discovery and personalized medicine, the success of the unavoidable filtering strategies of thousands variations lies in their implementation into convivial and versatile software tools. End users with no computational skill have to be autonomous to conduct and combine themselves different filtering approaches, depending on their assumptions and of their study design, leading them to extract a limited list of likely candidate genes underlying a genetic disease.

With this aim, in partnership with and for medical geneticists, we developed EVA (Exome Variation Analyzer), a user-friendly web-interfaced free software dedicated to filtering strategies for medical projects investigated with exome sequencing. EVA integrates the main filters dealing with common variations, molecular types, inheritance mode and multiple samples. Here we report a demonstrative case study with EVA that allowed to identify a new candidate gene related to a rare form of Alzeihmer disease [25]. We discuss our development choices and the position of EVA among other filtering tools recently published.

## Methods

### Implementation of EVA

ExomeDB was developed under MySQL (5.0). The main tables are Variation, Gene and Individual in which data are integrated from a list of variants (SNV, indel) associated with their annotations (*Cf*. Methods section, 'Data' subheadings). Currently, EVA works with the NCBI 37 (hg19) build version of the human genome but integrates an archive for the previous version (NCBI 36, hg18). Each new project is subject to a remote loading using an online *Variation integration *module that accepts TXT files and XLS files. The web interface was developed under PHP (5.3.2-1). For the implemented filtering strategies (*Cf*. Result section, 'Filtering strategy module' subheadings), a combination of criteria selected by the user, is transformed into an SQL query and sent to the ExomeDB database. Then, EVA's interface displays the remaining variations in table browsers (*Cf*. Result section, figures). The *Variation statistics *module proposes interactive bar and pie vizualizations of exome data implemented with the free JavaScript charting library Highcharts (Highsoft Solutions AS). To assure the confidentiality of the exome data, EVAs integrates an *Authentication *module requiring a login and a password given by an administrator. Each login/password is strickly project specific. Users can only see and manage their own exome projects. Some performance statistics are described in the Result section. At the time of this writing, EVA's interface is accessible at the web address http://plateforme-genomique-irib.univ-rouen.fr/EVA/index.php through the described authentication process. EVA's current and update versions will be freely available under a Creative Commons Attribution-NonCommercial-NoDerivs 3.0 Unported License (CC-BY-NC-ND) and will be downloaded from the same web site.

### Data

The input file (TXT file or XLS file) of the *Variation integration *module of EVA is a list of variants (SNV and indel) obtained from an independant variant calling procedure (briefly in this study: Solexa Illumina technology, base calling from raw image files with RTA1.8/SCS2.8, Illumina pipeline CASAVA 1.7 with ELAND v2) and then annotated (in this study: proprietary bioinformatics process from IntegraGen company, Genopole^® ^Evry, France, [26]). Although actually, the format of these files is a proprietary format, it includes classical annotations for the detected variations and the affected genes. For the detected variations main information are: the chromosome and the genomic position, the number of the read bases for each nucleotide, the reference base and the modified base deduced from an allelic count procedure and annotated with the genotype homozygosis or heterozygosis status, the number of total sequenced bases and the number of used bases for the detection variant, the score of the variation depending on the quality and coverage, the type of variation (SNV or indel), the rs name if known in dbSNP (in this study dbSNP131 [11], HapMap [27]), CIGAR format and length for the indels. For the affected genes the main information are: the gene name (NCBI GeneID), the NCBI RefSeq accession number [28] for all mRNA variants expressed by the gene, the type of affected position (exons, introns (only variations in +/- 20 regions are considered), 5' or 3' UTR) and the corresponding number of the exon or intron along the gene structure, the functional categories of variations (synonym, missense, stop loss and nonsense for SNV, frameshift or not for indel), the exon or intron start and stop positions included the variation and finally position of the variations in the corresponding protein sequence with the description of the codon and corresponding amino-acid for both the reference protein and the detected variations.

### Case study: the Alzheimer disease

Thanks to a nationwide recruitment (Clinical Research Hospital Program from the French Ministry of Health (GMAJ, PHRC 2008/067)), exome sequencing was performed in fourteen autosomal dominant early-onset Alzheimer disease (ADEOAD) unrelated index cases without mutation on known genes (*Amyloid precursor protein (APP), presenilin1 *and *2 (PSEN1 *and *2*)) and also without known copy number variants of *APP *gene and genes involved in Amyloid beta (Aβ) peptide processing or signaling. IntegraGen company (Genopole^® ^Evry, France, [26]) performed exome sequencing. Three micrograms of genomic DNA from each individual, extracted from peripheral blood lymphocytes and sheared by sonication to obtain an average fragment size of 150-200 bp, were used for the construction of a shotgun sequencing library using paired-end adapters. Exome capture was performed using the SureSelect Human All Exon kits 38 Mb version 1 (Agilent) (*n *= 12) or SureSelect Human All Exon kits 44 Mb version 2 (Agilent) for a second batch (*n *= 2).

Sequencing was realised on an Illumina Genome Analyser GAIIx (*n *= 12) or on an Illumina HiSeq 2000 (*n *= 2). Raw image files were processed by using the Illumina pipeline (RTA1.8/SCS2.8 and CASAVA 1.7). For the genetics variant detection, the 76 bp sequencing reads were aligned to the NCBI human reference genome (NCBI (*n *= 12) or NCBI 37 (*n *= 2)), using ELANDv2. Means coverage were of 65-fold (*n *= 12) and 80-fold (*n *= 2) with a percentage of aligned reads ranging between 88% and 95%.

Only high quality variations having a QPhred threshold > 10 were conserved (86% of the targeted bases). The annotation procedure of the detected variations only concerned those included in the coordinates given by the exon kits capture extended to +/- 20 pb in the flanking intron. The description of the annotated files is explained in the Methods section, 'Data' subheadings. Each annotated file corresponding to the project (14 individuals) was integrated in ExomeDB using the *Variation integration *module of EVA.

## Results

### Overview: ExomeDB and EVA web interface

**Figure 1:**
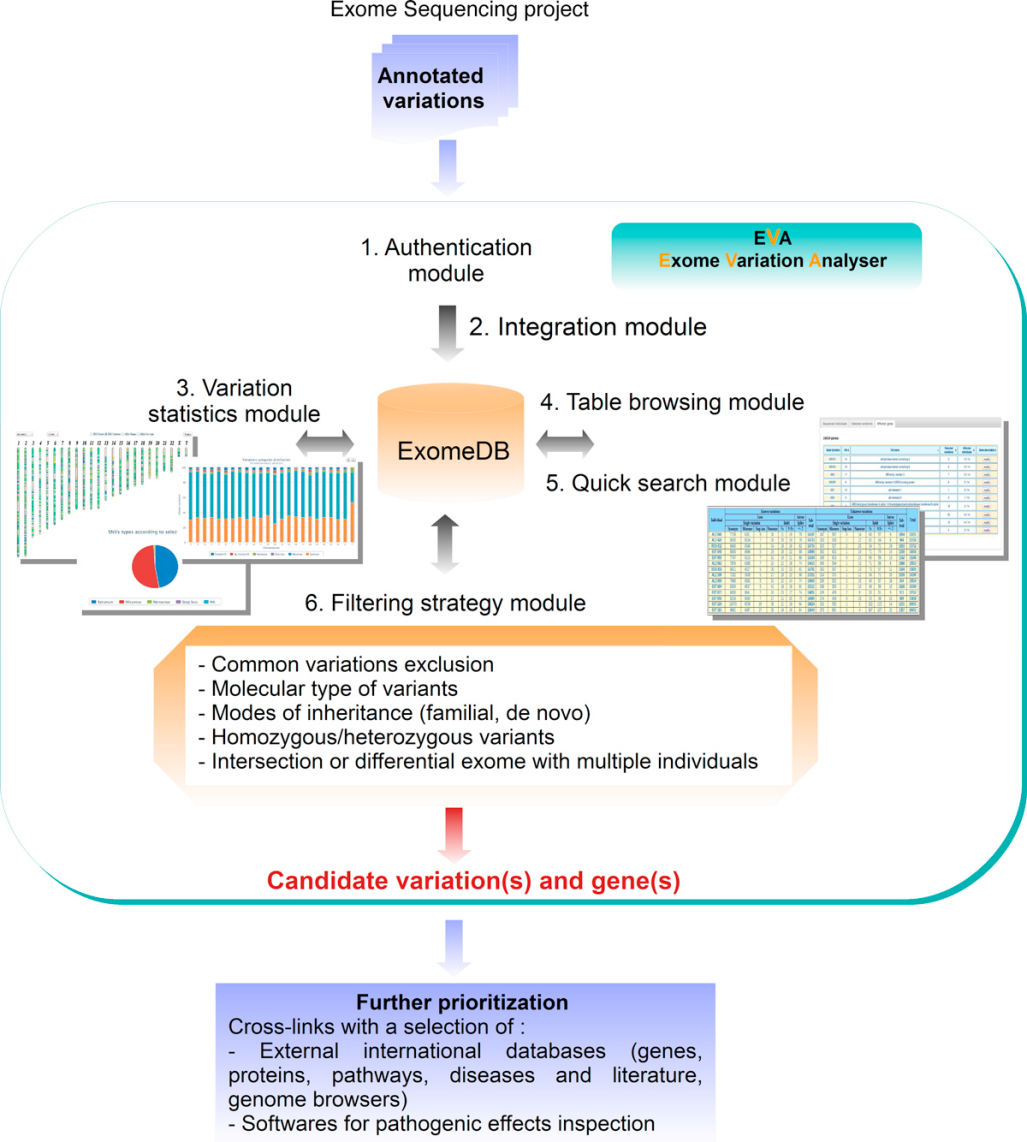
**Overview of EVA with the six exome mining modules**. To store and manage variation data, EVA consist of a database, ExomeDB and a friendly web interface. To date six modules permitted to authentify the user (1), to integrate data (2), to search and browse data with charts (3) or tables (4,5) and finally to conduct a customizable filtering strategy (6) by a combination of selected criteria dealing with common variations, molecular types of variants, inheritance modes and multiple samples intersection or difference.

For a given WES project, corresponding to several individuals and their respective variations, EVA manages data thanks to six modules (Figure 1). In input, after authentication (*Authentication module*), an online *Variation integration *module takes the variations files (for details of the format, Cf. Methods section, 'Data' subheadings) obtained from an independant variant calling bioinformatics pipeline. Annotated variations are stored in a relational database ExomeDB which main tables are the annotated variations, the corresponding genes and the individuals (Cf. Result section, 'performance'' subheadings').

**Figure 2:**
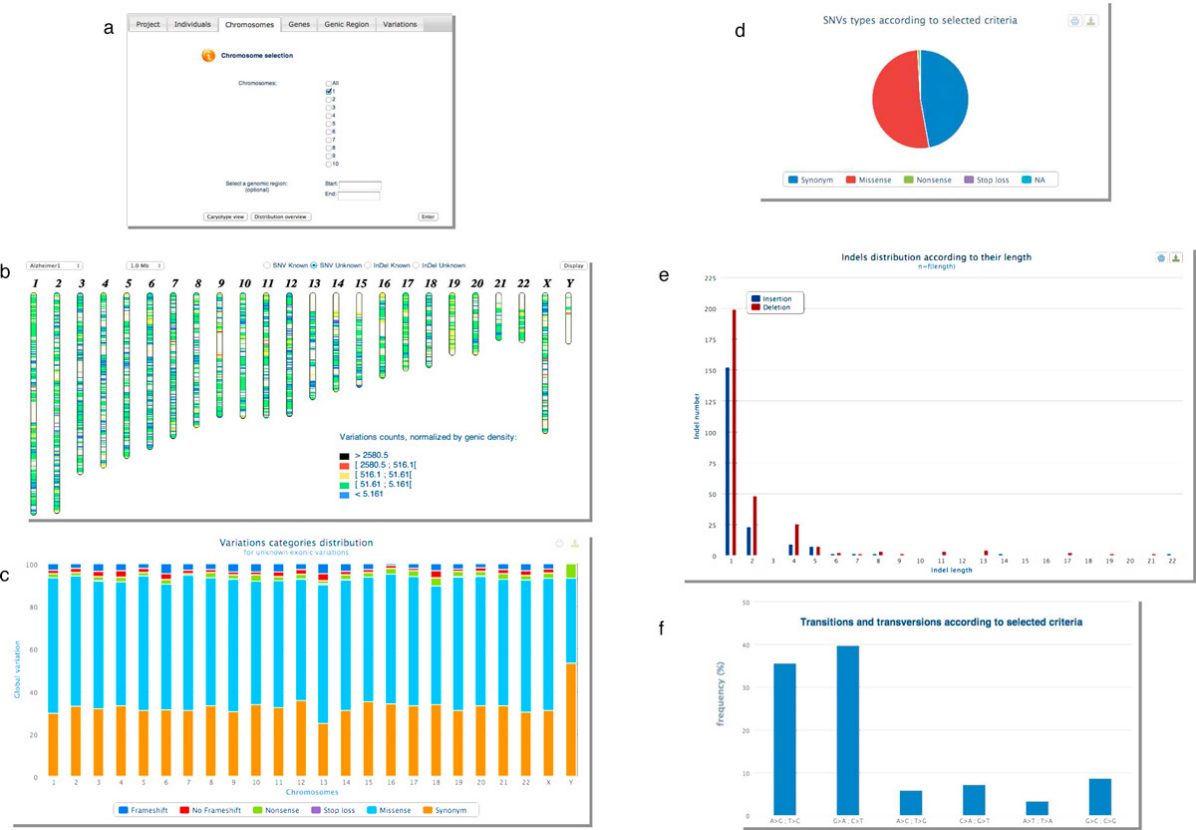
**Examples of graphical visualization with the *Variation statistics *module of EVA**. Through a guided mode selection (a), the distribution of variations for the 14 ADEOAD exome project are graphically represented by a caryotype view (b), a bar chart or a pie chart distribution (c, d). Useful analysis of indels (e), ratio of transition (purine/purine or pyrimidine/pyrimidine) substitutions to transversion (purine/pyrimidine) substitutions (f) and substitution matrix of amino-acid (not showed) are proposed.

**Figure 3:**
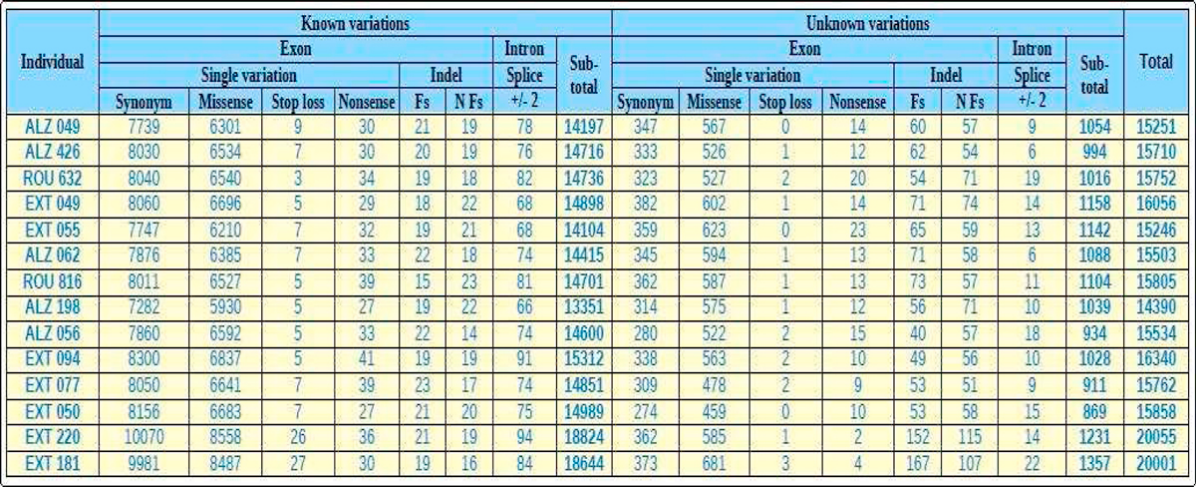
**Raw '*Variation overview*' in EVA for the 14 ADEOAD exome project**. Both individuals **EXT 220 **and **EXT 181 **belong to batch #2 described in the section 2.2, all the others belong to batch #1.

The web interface integrates four other modules for exome mining. The *Variation statistics *module allows through a guided mode selection of individuals, chromosomes, genomic regions, genes, genic region, type of SNV or indel, to summarize in tables and to graphically represent the global or selected distribution of variations of a given WES project (Figure 2). The *Table browser *module allows to precisely explore data by project, individual, gene or variation through rigorous and sortable categorized tables (Figure 3) (iii) *the Search *module can be used for a direct and quick access to a specific region, gene, variation for a given project, and finally (iv) the *Filtering strategy *module, which is the major element in exome mining to discover potential canditate genes, offers to combine filters for common variations, molecular types, inheritance mode and multiple samples to drastically narrow down variations (see details below). The selected combination is transformed into a SQL query and sent to the ExomeDB database.

**Figure 4:**
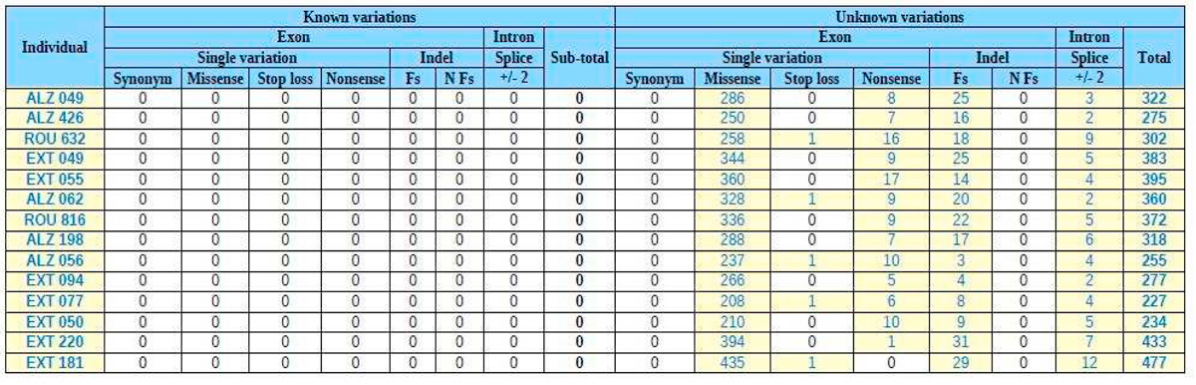
**Primary screened '*variation overview*' after filtering strategy functionality of EVA for the 14 ADEOAD exome project**. Both individuals **EXT 220 **and **EXT 181 **belong to batch #2 described in the section 2.2, all the others belong to batch #1.

**Figure 5:**
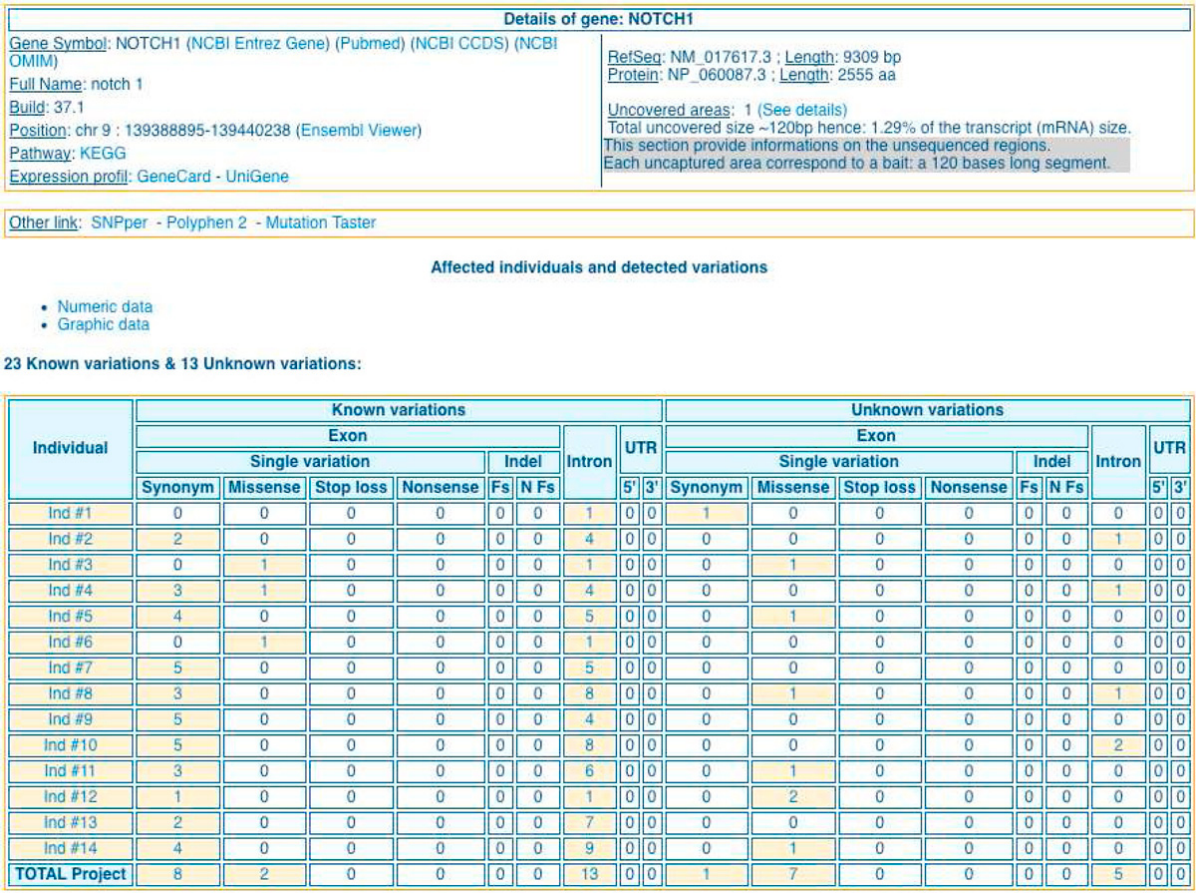
**'*Gene detail*s' table in EVA**. For a given gene (here NOTCH1) it is possible: (top) to get information about its chromosomic laction, links to useful public databases (Entrez, Pubmed, CCDS, OMIM), areas not captured during the pre sequencing protocol and links to interpretation tools (SNPper, Polyphen 2, Mutation Taster); (bottom): the categorized variations located in that gene for all the individuals of a project.

**Figure 6:**
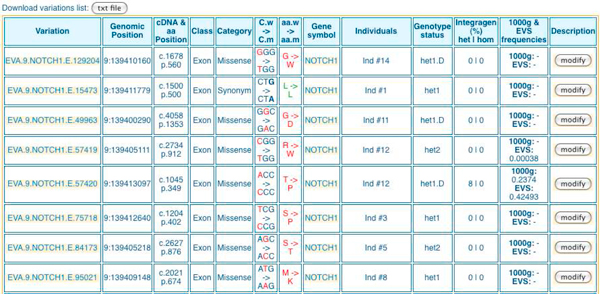
***'Variation list' *table in EVA**. For a given gene (here NOTCH1) it is possible to get the list of all the variations found in that gene for all the individuals of a project. For each variation the following information is listed: genomic position, gene position, functional class, category, wild codon vs modified codon, exome frequencies (heterozygous, homozygous), gene symbol, individual and genotype status.

**Figure 7:**
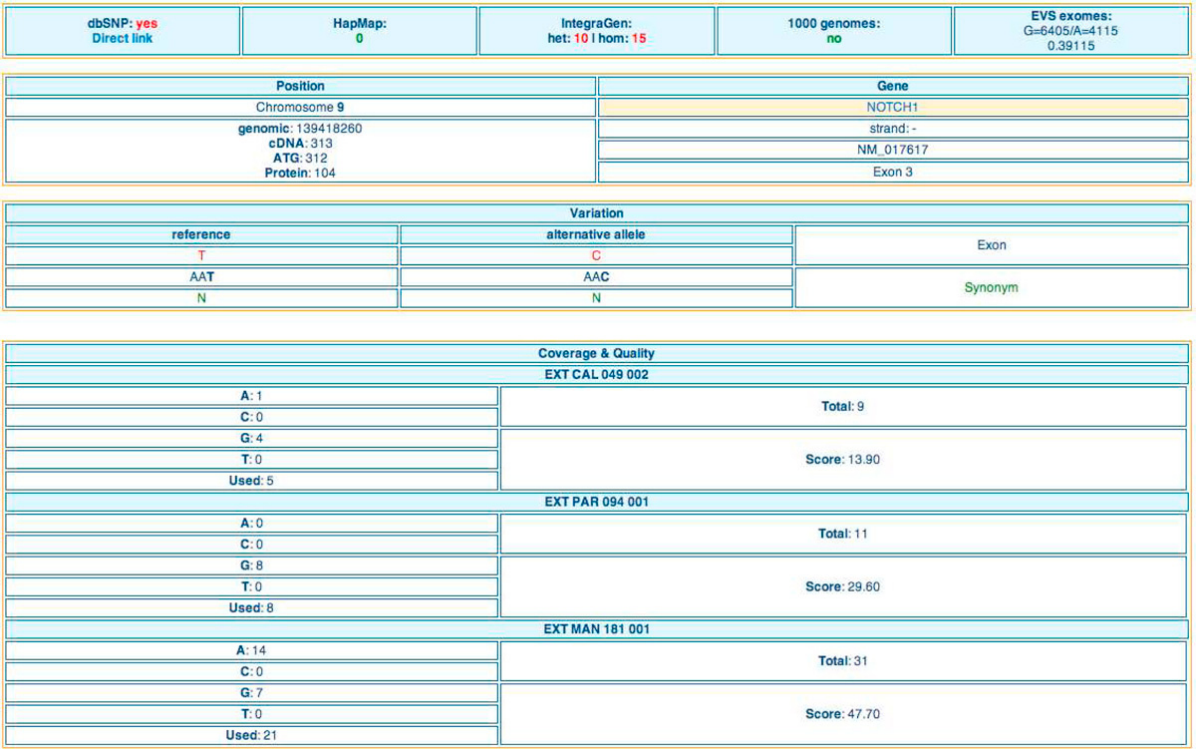
**'*Variation details' *table in EVA**. For a given variation, it is possible to get information such as: links to dbSNP, counts in HapMap, 1000 genomes and EVA, genomic position, gene information, coverage and quality per individual.

Query results of the *Table browser *module, *Quick search *module and *Filtering strategy *module can be explored by five elements types presented in interactive tables: '*variation overview' *(Figure 3 and Figure 4*), 'gene list', 'gene details'* (Figure 5), *'variation list'* (Figure 6) and *'variation details'* (Figure 7).

In the 'variation overview' tables (Figure 3), the set of all the variations is divided in known and unknown variations according to the information in dbSNP. Due to the molecular process of the exome capture kit, most variations occur in exons but some detected variations also occur in splice sites. Even if ExomeDB integrates variations extended to +/- 20 pb in the flanking intron, we choose to show on the table only variations extended to +/- 2 pb in the intron, corresponding to the dinucleotide splicing site. Variations in exons can be SNV or indels. We categorized single variations into four functional classes: synonymous, miss sense, stop loss and non sense. For indels we classified into two categories: frameshift or non frameshift.

In output, EVA offers *export files (*CSV for tables, various graphical formats for the *Variation statistics *module). EVA also provides several cross-links with a selection of relevant external international databases and softwares for further functional and pathogenic effect inspection of the sorting variation and gene candidates (see details below and on Figure 5).

### Filtering strategy module

The *Filtering strategy *module integrates the current main categories of filters based on common variations, molecular type of the variants, modes of inheritance, homozygous or heterozygous nature of the allelic variant and multiple individuals.

First, EVA compares the data to international catalogues of variations. In a constitutive sorting, the set of all the variations is divided in known and unknown variations according to the information in the dbSNP (Figure 3 and Figure 4). EVA also offers to reduce the number of variations by confronting them to the HapMap Project [27], the 1000 genomes Project [10], Complete Genomics public data [29], IntegraGen public data [26] or the Exome Sequencing Project [30]. In addition, other filters or table browsers offer to sift variations depending on their: (i) functional categories for SNV (synonymous, miss sense, stop loss and non sense) and indels (frameshift or non frameshift); (ii) genic region (UTR, CDS, intronic splice region) or genomic region; (iii) quality score and coverage. Finally, one of the strengths of EVA is the implementation of inheritance filters considering intersection or conversely differential exome strategies: (i) recurrence strategy for dominant or recessive independent familial cases (filters select the genes the most affected by remaining variations among a specified number of non related individuals.); (ii) filters for homozygous, heterozygous or composite cases in intra-familial studies (filters extract genes with remaining common variants among selected related individuals); (iii) and *de novo *strategy for sporadic cases (filters select genes with remaining variations found in a diseased child but not in the two healthy parents (sporadic case, trio-family).

For each strategy the displayed result is a list of potential candidate genes associated with the number of affected individuals (*'genes list'*). Again, it consists on an interactive table that could be readily explored. The user can get '*gene details*' (Figure 5) containing interactive links to other tables '*variation overview' *(Figure 3), *'variation list' *(Figure 6), and '*variation details*' (Figure 7). To ensure a rapid execution of EVA (Cf. 'Performance' subheadings) implemented in priority to focus on filtering strategies, we made the choice not to include variant effect prediction functionalities. Nevertheless, to facilitate the further prioritization of remained variations and genes, external functional and pathogenicity interpretation tools (SNPper [31], Polyphen 2 [32], MutationTaster [33]) are cross-linked as well as useful external international databases of genes, proteins, pathways, diseases and literature and genome browsers.

### Case study: Alzheimer disease

After screening more than one hundred autosomal dominant early-onset Alzheimer disease (ADEOAD) families for known mutations (*Cf*. Methods section, 'Case study' subheadings) the molecular basis of this rare disorder still remained unexplained in several of them. Moreover, the lack of DNA for affected relatives precluding a linkage analysis in these cases, a full exome sequencing strategy was decided to identify new candidate gene(s) with unknown mutations. Exome sequencing, variation detection and annotation were performed by IntegraGen company (Cf. Methods section, 'Case study' subheadings) for fourteen ADEOAD unrelated index cases. The annotated variant files were subjected to ExomeDB to a remote loading using the online *Variation integration module *of EVA. Then, the intersection recurrence filtering strategy was applied with EVA. Here the main steps of our filtering procedure are summarized:

Firstly we displayed the full project data. Figure 1 corresponds to the raw '*variation overview' *of this exome project integrated in EVA and is obtained with the *Table browser *module. In this interactive table, variations are displayed by individuals and divided into two groups on the dbSNP131 referencing basis. '*Known*' means variations referenced in dbSNP, while '*unknown*' means variations not referenced in dbSNP. Within those groups the variations are rigourously and usefully displayed by two functional classes '*Exon*' and '*Intron*' *(*only two intronic base pairs before and after exons ('*+/-2*')). Exonic variations are classified into six sub-categories, '*Synonym*', '*Missense*', *'Stop loss*' and '*Nonsense*' for SNV and Frameshift ('*Fs*') and No Frameshift ('*Nfs*') for indels. In total, 14,390 (batch #1) to 20,055 (batch #2) genetic variants were identified *per *exome according to the capture protocol (15,600 in average for batch #1 and 20,028 in average for batch #2). Among these, 6.6% in average are unknown variations (1028 in average for batch #1 and 1294 in average for batch #2).

Secondly, thanks to the *Filtering strategy *module we applied a stringent primary screening based on [common variations + molecular type of variants + heterozygous nature]. Figure 2 corresponds to the *'variation overview' *after this one: variations retained were previously '*unknown*' *(filtered against db SNP31)* but then filtered against HapMap exome projects, and against 42 IntegraGen exome projects from unrelated individuals with non-neurodegenerative diseases, the other filters parameters were '*non-synonym*' *SNV, 'frameshift coding'* indels, *'splice acceptor and donor site' *and *'heterozygous'*. Finally, the number of unknown variations by individual drastically decreases from 1028 in average for batch #1 and 1294 in average for batch #2, to 310 and 455 respectively. So, remaining unknown variations after this primary screening with EVA represented only 2% of total genetic variants identified *per *exome *versus *6.6% in the raw data.

Thirdly, a secondary screening of the remaining variations based on the inheritance mode assumption of the disease was applied with an intersection recurrence procedure. Table 1 summarizes the number of genes harboring at least one of these variants classified according to their recurrence in the patient sample. The 14 patients did not have in common a single altered gene, indicating that, within this sample, the disease was genetically heterogeneous. Nevertheless, we observed that the number of candidate genes drastically decreased with the increasing number of concerned individuals. So, EVA enabled geneticists to focus further investigations on the affected genes shared by a minimum of 5 patients, representing a short list of less than 10 genes.

**Table 1:** Secondary screening obtained thanks to the 'recurrence' filtering Strategy functionality of EVA for the 14 ADEOAD exome project.

Number of individuals	Number of genes with remaining variations
14/14	0
13/14	0
12/14	0
11/14	0
10/14	0
09/14	0
08/14	1
07/14	3
06/14	3
05/14	7
04/14	31
03/14	112
02/14	542
01/14	2730

Finally, after wet investigations (Sanger resequencing verifications, family co-segregation analysis, genotyping of each variant in 1500 control individuals, RT-PCR expression analysis) combined with *in silico *analysis (predicted functional impact of each variation, comparison to the data set from the 1000 genomes project [10], and from Complete Genomics [29]), one gene (*SORL1*) containing unknown mutations in 5/14 exomes (nonsense (*n* = 1) or missense (*n* = 4)) has become a new strong candidate gene for the ADEOAD [25].

### Performance

To date, ExomeDB stores WGS projects (multiple unrelated cases, duo or trio cases) corresponding to a total of 23 individuals and contains also targeted resequencing projects corresponding to 5 genes for 25 individuals. As showed on Table 2, the size of ExomeDB is about 400 Mb, mainly due to the tables Variation (112.25 Mb) and Individual_Variation (271.09 Mb). Tests of EVA have been performed on the ADEOAD exome project (14 individuals) with one user logged in. The server is running Linux with four 3 GHz processors, 5 GB RAM and 150 GB HD. We use the "mysqli::prepare" mode which speeds up the request time once the first request has been treated. Table 3 shows request times in both cases. While it is clear that performances depend on the number of users logged in simultaneously and on the number of variants in the database (177,303 currently), EVA works with a reasonable time of execution compatible with the regular needs of a medical genetics laboratory.

**Table 2:** Performance of EVA: Tables size of ExomeDB

Tables	Size (Mb)
Gene	7.03
IG_NoCouv	5.55
Individual	0.02
Individual_Variation	271.09
Project	0.02
Project_Individual	0.05
User	0.02
User_Project	0.06
Variation	112.25

**Table 3:** Performance of EVA: Running times of EVA modules

Request	Time (1st time)	Time (after)
Table Browse	15 s	3 s
Quick search	9 s	1 s
Filters loading	13 s	2 s
Filters execution	7 s	1 s

Tests of EVA have been performed on the ADEOAD exome project (14 individuals) with one user logged in.

## Conclusions

EVA is developed to be a user-friendly, versatile, efficient-filtering and free assisting software for whole exome sequencing, providing a response to new needs at the expanding era of medical genomics investigated by these targeted next-generation sequencing technologies, for fundamental research, clinical diagnostics and personalized medicine [12,14-16,19,21,24]. Interfacing various now commonly adopted filtering criteria and strategies on whole exome data, EVA thereby makes non-programmer medical geneticists autonomous to pinpoint themselves among ~20,000 variations per individual exome, few candidate variations and genes related to a rare disease, depending of their specific assumptions and study design.

EVA constitutes a platform for exome sequencing data storage and for drastic screening of clinical relevant genetics variations. Thanks to different modules (i) it integrates and stores annotated exome variation data as strictly confidential to the project owner, (ii) for the analytical process, it proposes to combine the main filters dealing with common human variations (various international external public data [10,11,26,27,29,30], molecular types and functional categories (synonym, missense, stop loss and nonsense for SNV, frameshift or not for indel; genic region i.e. UTR, CDS, splice site), homozygous or heterozygous nature of the allelic variant, inheritance modes and multiple samples considering intersection or conversely differential exome strategies (independent familial cases, intra-familial studies, sporadic cases), quality of the variations (iii) it offers quick searching or advanced browsing of annotated data and filtered results thanks to various interactive categorized or sortable tables and useful graphical visualizations (iv) finally it offers export files and cross-links to external relevant databases and softwares for further functional effects inspection [31-33] of the small subset of sorted candidate variations and genes.

EVA has been used to successfully identify a new candidate gene, *SORL1*, related to a rare form of Alzeihmer Disease (ADEOAD), despite a genetics heterogeneity [25]. *SORL1 *encodes the Sortilin-related receptor LR11/SorLA, a protein involved in the control of amyloid beta peptide production, the same pathway as previously known genes *APP*, and *presenilin 1 and 2*. In this case study, the primary screening with EVA (based on the mutation types and common human variations) reduced unknown variations to only 2% (330 on average) of total genetic variants identified per exome. The secondary screening implementing the intersection recurrence strategy led to a short list of genes (< 10) on which geneticists focused for further *in silico* and wet experiments and among which they discovered one. In 5 patients of the 14 independant index cases investigated, we found that the *SORL1 *gene harbored unknown nonsense (*n* = 1) or missense (*n* = 4) mutations.

Performance tests showed that EVA run with a reasonable time of execution compatible with the regular needs of a medical genetics laboratory. For the case study it takes between 21 s (1^st ^time) to 3 s (after) to load and execute the selected filters (server with four 3 GHz processors, 5 GB RAM and 150 GB HD, and with one user logged in) from the currently 400 MB size of ExomeDB.

The commonly assumption for WES mining is that causal variants related to a Mendelian disorder under investigation will not be present in public databases of genetic variations or other exome sequencing projects [1,13,14,17,19,22-24]. That is why, the more variation data available the more the filtering strategies in exome mining would be successful. To enhance its filtering performances, EVA confronts exome data currently to 6 external public data [10,11,26,27,29,30] and will be regularly updating as new large-scale variations data will be published.

Some polymorphisms of these ressources (dbSNP) are not associated with their allelic frequency and lack experimental annotation of their functional impact. So, projects like the SNP database of effects (SNPdbe) [34], storing computationally annotated functional impacts of non synonymous SNPs or the annotation of 1000 top human cancer genes frequently mutated [35] could be of interest for EVA improvement.

Alternative tools designed for the similar task as EVA have been recently published [35-38]. Varsifter [36] is a graphical Java program for desktop computers. It is designed to read exome-scale variation data in either a tab-delimited text file with header, or an uncompressed VCF file. It proposes numerous filtering options but doesn't propose graphic visualization nor statistical summaries of a WES project. SVA [36] is largely based on a genome browser to deal with WGE as well as WES and sifts small and large variants. While it proposes many manipulations of data, it is not clear if inherihance filtering are implemented. More, SVA is a JAVA program requiring a recommend hardware equipped with at least 48 GB of RAM and 1TB of free hard disk, which are substantial computational resources, in practice not very compatible to all individual laboratory. Finally, VAR-MD [37] is a family based tool. It analyzes WGS and WES variants exclusively in small human pedigrees with Mendelian inheritance excluding the scope of the differential exome analysis.

As perspectives are concerned, the input format for EVA for the *Variation integration *module, which is currently a proprietary format will be soon standardized in order to offer a wide use of this tool; we retained the Variant Call Format (VCF) format, generated by the 1000 Genomes Project. The *Variation integration *modulewill also allow the annotation of the raw variations by both Annovar [39] and the Variant Effect Predictor Ensembl API [40]. Regular updates are made concerning build version of the human genome, international variation catalogues and improvement of filtering functionalities as well as organization of results tables and graphics. Future developments include a graphical representation of a candidat gene with its variations and a more specific filtering strategy for somatic mutations.

## List of abbreviations used

ADEOAD: Autosomal Dominant Early-Onset Alzheimer Disease; WGS: whole genome sequencing; WES: whole exome sequencing; SNV: single nucleotide variation.

## Competing interests

The authors declare that they have no competing interests.

## Authors' contributions

DC expressed the need of the tools, supervised the project and its design, oriented the filtering strategy functionality and realized the case study. SC realized the implementation of EVA, co-realized the case study, contributed to the manuscript. AL, ML, EPG and TL co-supervised the implementation of ExomeDB and the EVA interface and contributed to the manuscript. CC implemented the *Variation statistics *module and contributed to the manuscript. HD co-supervised the project and its design, oriented the categorization of variations and the different views of results on interface. HD and TL coordonned the collaboration between the two teams INSERM U1079 (clinicians) and TIBS-LITIS (bioinformaticians) and wrote the paper.
